# Understanding competing discourses as a basis for promoting equity in primary health care

**DOI:** 10.1186/s12913-019-4602-3

**Published:** 2019-10-28

**Authors:** Amélie Blanchet Garneau, Annette J. Browne, Colleen Varcoe

**Affiliations:** 1 Faculté des sciences infirmières, Université de Montréal, C.P. 6128 succ. Centre-Ville, Montréal, QC H3C 3J7 Canada; 2 School of Nursing, University of British Columbia, T201- 2211 Wesbrook Mall, Vancouver, BC V7C 5S1 Canada

**Keywords:** Health equity, Primary health care, Discourses, Structural inequities, Efficiency, Racism, Discrimination

## Abstract

**Background:**

Globally, health inequities persist with effects on whole populations and the most profound effects on populations marginalized by poverty, discrimination and other forms of disadvantage. In the current neoliberal political-economic context, health inequities are produced and sustained by the inequitable distribution of social determinants of health and structural inequities such as discrimination and institutional racism. Even in the context of healthcare organizations with an explicit commitment to health equity, multiple intersecting discourses, such as ongoing efficiency discourses, and culturalist and racialized discourses, are in constant interaction with healthcare practices at the point of care and the organizational level, limiting providers’ and organizations’ capacities to address structural inequities. Attention to discourses that sustain inequities in health care is required to mitigate health inequities and related power differentials. In this paper, we present findings from a critical analysis of the relations among multiple discourses and healthcare practices within four Canadian primary health care clinics that have an explicit commitment to health equity.

**Methods:**

Informed by critical theoretical perspectives and critical discourse analysis principles, we conducted an analysis of 31 in-depth interviews with clinic staff members. The analysis focused on the relations among discourses and healthcare practices, the ways in which competing discourses influence, reinforce, and challenge current practices, and how understanding these dynamics can be enlisted to promote health equity.

**Results:**

We articulate the findings through three interrelated themes: equity-mandated organizations are positioned as the “other” in the health care system; discourses align with structures and policies to position equity at the margins of health care; staff and organizations navigate competing discourses through hybrid approaches to care.

**Conclusions:**

This study points to the ways in which multiple discourses interact with healthcare organizations’ and providers’ practices and highlights the importance of structural changes at the systemic level to foster health equity at the point of care.

## Background

The rhetoric of health equity has been adopted globally and presented in key publications such as Integrating Social Determinants of Health and Health Equity Into Canadian Public Health Practice [1] and Achieving Health Equity: A Guide for Health Care Organizations [2]. These reports have specifically called for bridging the knowledge to action gap for health equity gains. Health equity, informed by social justice aims, focuses on being responsive to people’s intersecting health and social needs, including paying particular attention to those at greatest risk of poor health. Health equity differs from health equality, a principle guiding most Western countries’ healthcare systems, which focuses primarily on offering equal access to health care, implying that people generally have similar capacity to obtain health care.

Strategies aimed to address health inequities in the health sector frequently target healthcare providers through educational and training programs, such as the development of cultural competence and, more recently, cultural safety, without necessarily involving broader organizational and structural changes [3–5]. Studies have shown that, in spite of the training provided to health care providers and their commitment to health equity, they remain influenced by pervasive, dominant social discourses [6–10]. These dominant discourses often reflect erroneous assumptions about the root causes of ill health, individualistic ideas of risk and risk management and individual responsibility, taken for granted assumptions about the importance of efficiency over effectiveness, and the inevitability of health and social inequities as a function of poor personal choices. Thus, because complex dynamics shape healthcare providers practices within organizations, education strategies are not enough to induce sustainable changes of practice. A process of organizational change involving changes in policies and processes is thus necessary so that healthcare providers move beyond shifting individual practices toward addressing structural inequities [4, 11].

Although knowledge and effective interventions at the organizational level to address health inequities have been documented, there is currently limited awareness and use of this knowledge [12]. There is also a lack of attention to how to operationalize equity as a priority. This lack of understanding about “what to do” about widening health inequities mirrors broader dominant discourses at the policy level in health care and in society. Thus, even in the context of organizations with an explicit commitment to health equity, multiple intersecting and often competing discourses shape healthcare practices at the point of care and the organizational level, limiting providers’ and organizations’ capacities to address structural inequities [13].

In this paper, we present the findings of a critical analysis of the relations among multiple discourses that are pervasive in health care, and health care providers’ perspectives and practices within four primary health care (PHC) clinics that have an explicit philosophical commitment to health equity. Through this analysis, we offer some strategies to make visible competing discourses in healthcare and reconcile them in the service of health equity.

### Dominant discourses in the health sector

Discourses are manifestations of ideologies and form individual and collective consciousness, which influence people’s actions [14]. In the health sector, liberal individualism is integral to dominant discourses that align with efficiency, egalitarian, culturalist, and racializing discourses, all of which can challenge the goal of equity [6, 15, 16]. Indeed, healthcare systems in most Western nations are located in a liberal political and economic ideology linked to the main tenets of capitalism. With its emphasis on marketization and competition, liberal individualism, a liberal ideology, has highlighted the importance of individual choice and productivity in the context of healthcare and health systems globally, even in the context of publicly funded healthcare systems [6]. In recent years, improving performance has then become an imperative in most Western countries’ healthcare systems [17]. For example, recent United Kingdom’s National Health Services (NHS) reforms have promoted improved healthcare productivity as a fundamental objective of policy and professional work. Reports show that instead of creating a better environment for care, this competitive, business-focused philosophy has led organizations to become focused only on output and not on the population needs [18].

#### Dominance of ‘efficiency’ and ‘equality’ discourses

At the point of care, efficiency discourses have shaped the language used by healthcare providers and placed emphasis on time pressures, care processes, and organizational tensions in a way that can compromise best practice and contribute to the entrenchment of a production-line mentality [7, 8].

For example, in a study aimed at examining the impact of a policy initiated to reduce wait times in Emergency Departments (ED) on clinical practice and medical trainee education, Webster et al. (2015) concluded that the emphasis on wait times resulted in more importance being placed on “getting the patient out” of the ED than on providing safe, person-centred medical care. The pressure to reduce wait times also meant fewer opportunities for teaching, resulting in medical staff both explicitly and implicitly teaching trainees that productivity in terms of processing patients as swiftly as possible is the top priority in their practice. This focus on processing patients as quickly as possibly – which is often constructed as “efficiency” – has also led healthcare providers to describe patients in terms that suggest that patients are the source of the problem despite underlying systemic issues [8]. Some examples are the use of terms such as *frequent fliers*, for patients who seek care at the hospital often; *bed blockers*, for patients who cannot be treated and discharged quickly; and *failure to cope*, for patients with social needs. Hence, delivering good quality care has been redefined in terms of managing and processing patients to reach targets among resource shortages, including time [7, 8]. Echoing earlier analyses [e.g. 19, 20], Moffatt, Martin [21] add that a new professionalism has arisen from this kind of efficiency discourse, reconstructing the professional responsibility of healthcare providers at the point of care. Productivity is now identified as an individualized professional duty as healthcare providers are seen not only as part of the problem of productivity but also its potential solution [21]. Thus, when efficiency is enacted to fulfill external fiscal pressures and incentives, it can shift the clinical focus away from the patient and toward the achievement of system efficiency [8], focusing on short-term gains (e.g. reduced length of stay) at the expense of longer-term concerns (e.g. readmission rates).

Equality is another principle guiding most Western countries healthcare systems. Going hand in hand with a liberal ideology, egalitarian (also known as equality) discourses reinforce beliefs about the equal agency of all individuals, expecting them to be responsible for their social position and behaviour regardless of their circumstances [22]. In Canada, for example, it is widely accepted that all citizens and landed immigrants have an equal opportunity to achieve optimum health because healthcare is a legislated social policy, publicly funded, and a guaranteed right [15]. Hence, the ideal of equality is often operationalized at the point of care with “equal treatment” in the context of presumed “equal opportunity” with providers aiming to “treat everyone the same.” However, evidence shows that the life expectancy of Indigenous people and low-income Canadians remains lower than the life expectancy of the general population [23]. Mortality rates and the prevalence of cardiovascular disease, heart disease, and diabetes are also higher among these groups. People living in rural and remote areas face challenges in accessing healthcare and are more likely to experience poorer health outcomes [23]. Consequently, while equality and fairness are seen as important professional values, treating everyone the same regardless of their circumstances further perpetuates inequity by obscuring the unequal power relationships between patients and providers and by ignoring structural and social inequities shaping people’s health [10, 13, 15]. Equality and personal responsibility, as central tenets of liberal individualism, are still taught to health sciences students as foundational axioms in most Canadian programs.

#### Pervasiveness of culturalist and racializing discourses

Within most healthcare contexts, attention is also paid to diversity. In concert with individualism and egalitarianism, culturalist and racializing discourses emphasize simplified and stereotypical representations of cultural and racialized groups [24]. Culturalism is “a form of stereotyping whereby culture, defined very narrowly and often in stereotypical ways, becomes the primary explanation for why certain groups of people may be experiencing particular health or social issues” [13]. Evidence also suggests that racialized ideologies and practices affect not only the administration of healthcare services but also the delivery of services to individuals and the allocation of resources, training and educational programs [15]. These dynamics of individual and institutional racism and culturalism are reflected in the policies and practices of mainstream healthcare organizations. Even in countries committed to democratic principles such as justice, equality, and fairness, negative feelings about minority groups, differential treatment, racism and other forms of discrimination coexist with these principles [15]. One consequence of these conflicting positions is a prevailing lack of support for policies and practices changing the cultural, social, economic and political order to address inequities. Furthermore, these policies and practices, because they challenge status quo conditions, are perceived to be in conflict, if not threatening, to liberal democracy [15]. For example, in Canada, the assumption is that because Canada is a society that endorses the principles of a liberal democracy, it could not sustain inequities, racism, and discrimination. Hence, when racism is shown to exist, it is explained as an individual-level issue, pre-empting questioning of the structural processes in place that could reproduce and sustain it. Giroux [25] argues that neoliberalism (contemporary liberal individualism) is central to the production and reproduction of racism and other inequities in Canada, because of the focus on individual freedom and choice to the detriment of notions of public good, civic responsibility and social justice, and the favouring of individual solutions to public issues such as poverty, lack of housing, and underemployment of racialized peoples.

#### Health equity discourses

Although dominant discourses may act as barriers to the implementation of equity principles at the point of care, varied notions of social justice and equity also influence healthcare practitioners’ training and day-to-day practice [26]. The mainstream sector has made efforts toward supporting health equity through research and practice related to patient-centred care, diversity, and cultural sensitivity in healthcare [27–29]. However, efficiency discourses often undermine such efforts.

Health equity initiatives tend to be isolated from the mainstream delivery system [30] and little is known about the challenges faced by organizations and their staff, and the strategies they use to strive to provide equitable care in the context of multiple competing discourses in health. Thus, in this era of mandating organizations to provide equity-oriented health care, it becomes even more important to make visible competing discourses in healthcare. Attention to the types of language and discourses that create and sustain inequities, and undermine equity-oriented efforts in health care, or counter such dynamics, is required to address and mitigate health inequities and related power differentials. Examining how multiple discourses interact at the point of care can help identify challenges and opportunities for promoting equity in healthcare.

## Method

The findings discussed in this paper are based on a secondary analysis of data from a larger study aimed at improving the capacity of organizations to provide equity-oriented health care (EOHC) by modifying structures, policies, and practices to address structural inequities. EOHC aims to mitigate the impacts of the ongoing effects of trauma and violence, the multiple and intersecting forms of discrimination and stigma, and the unfair distribution of the social determinants of health that sustain social and health inequities. To that purpose, an innovative multi-component, organizational-level intervention known as ‘Equipping Health Care for Equity’ (EQUIP), was designed, implemented and evaluated in partnership with four primary health care (PHC) clinics in two provinces in Canada [31–33]. The clinics provide services to between 1300 and 3700 individuals per clinic. The majority of clients experience challenges accessing care and are significantly affected by structural inequities, including systemic racism, poverty and gender-based violence. In EQUIP, EOHC is conceptualized as comprising the following interrelated key dimensions: trauma-and-violence informed care, culturally safe care, and harm reduction, tailored to fit the unique contexts and organizational settings in which they are implemented [32]. These key dimensions of EOHC provided the basis for the EQUIP intervention, and are described in detail in prior publications [32, 33]. In brief, the EQUIP intervention involved two main approaches: (a) staff education and integration discussions delivered both face to face and through online modules, and (b) a process of organizational integration and tailoring, which each clinic led and subsequently implemented to address specific priorities they identified.

We analyzed data from in-depth individual interviews with 31 clinic staff members conducted as part of the EQUIP research in the context of long-term research relationships (3–15 years), delivery of the intervention over a 2-year period, and the collection of observational data throughout. Participants were purposefully selected to represent a diversity of roles and experience at the clinics. Participants included pharmacists, physicians, nurses, nurse practitioners, dieticians, medical office assistants (MOA), substance use counsellors, social workers, outreach workers, and administrative leaders. The length of employment varied from 2 months to 15 years, with a mean of 7 years. Most interviews were conducted by principal investigators, including second and third authors, who have extensive experience in qualitative interviewing. Trained research staff conducted some interviews. Interviews lasted from 30 to 60 min. They were recorded, transcribed, and all identifying information was removed before analysis.

Critical theoretical perspectives and the principles of Fairclough’s [34] critical discourse analysis (CDA) informed our approach. Critical perspectives provide a theoretical lens through which to look at power dynamics reflected in participants’ discourses, and the sociopolitical contexts and discourses that shape healthcare providers’ perspectives and practices [35]. CDA combines a critique of discourse and an explanation of how it figures within and contributes to existing social reality, as a basis for action to change that existing reality [34]. One of the aims of CDA is to identify how ideology and power operate within discourses. Thus, CDA principles are suitable to generating a deep understanding of competing discourses influencing healthcare practices, and making power relationships explicit. We focused the analysis on the relations among discourses and healthcare practices, the ways competing discourses influence, reinforce, and challenge current practices, and how understanding these dynamics can be enlisted to promote health equity.

Fairclough (2015) proposes an analytic framework for exploring the relationships between text and its social context which comprises three interdependent dimensions of discourse: text, discourse practice, and sociocultural practice. Fairclough suggests approaching the analysis of the dimensions simultaneously. It is in the interconnections of text, discourse practice and sociocultural practices that patterns and disjunctions can be highlighted and explained. First, we read all interviews and selected specific sections to analyze. We used NVivo™ to organize and code the data. We selected sections where the participants described their approach to care, perceptions of their roles and relations with patients and other staff. We then analyzed the selected sections in a non-linear manner keeping in mind Fairclough’s three interdependent dimensions of discourse to identify the challenges staff members face at the point of care and the strategies they use to engage with multiple discourses and internalize or resist these discourses in practice. Beginning with a textual analysis (text), we paid attention to participants’ word choice, lexicalization, ideological orientation, taken-for-granted assumptions, contradictions, and descriptions of power relations. We then moved to analyzing the process of production and reception of the text (discursive practice) and its interactions with power structures (sociocultural practice). We examined how various discourses figure in the establishment, reproduction and shifts in power relations at the clinic and systemic levels, and how they might be enlisted to promote health equity. Co-authors met regularly to ensure consistency between the data collected and the emerging themes and descriptions. An audit trail of analytical insights was kept throughout the analysis.

## Results

The analysis illustrated that discourses dominant in the broader Canadian health sector were readily reflected in the language and practice of clinic staff. Some of the discourses impeded efforts toward EOHC and contributed to tensions in operationalizing the clinics’ and practitioners’ stated values, aims, and philosophies. We identified three interrelated themes representing the relations among broader discourses in health and health care practices: 1) equity-mandated organizations are positioned as the “other” in the health care system, 2) discourses align with structures and policies to position equity at the margins of health care, and 3) staff and organizations navigate competing discourses through hybrid approaches to care. Equity-mandated organizations are often excluded from the mainstream healthcare system in material and professional ways, creating pressures on healthcare providers at the point of care. Operating at the margins of healthcare influences how practice is structured, and how healthcare providers’ practice, which contributes to positioning equity as outside of usual practice frameworks. Because equity is positioned at the margins of health care, organizations and providers must navigate dominant discourses, such as efficiency and culturalist and racializing discourses, through hybrid approaches to care, sometimes by harnessing these discourses in the service of EOHC.

### Equity-mandated organizations are positioned as the “other” in the health care system

Participants expressed concerns about being perceived by other providers in the local community as outsiders, working at the margins of the healthcare system, resulting in some participants’ sense of operating in isolation and feeling excluded from mainstream healthcare system. This sense of exclusion was evident as participants referred to the mainstream system as the “outside world” (nurse practitioner) and “out there” (social worker). They also contrasted the populations they serve at the clinic from the population served in the mainstream healthcare system. Services offered at the clinics target underserved populations who face many challenges related to their health status, socioeconomic conditions, and ability to access good quality healthcare. For example, one participant described the “special” population they served contrasting with the “general” or “average” population (pharmacist).

Participants felt marginalized in both professional and material ways, reflecting a sense of exclusion. Participants repeatedly stressed the uniqueness of the services they provided and the impossibility of their clientele receiving care from the mainstream healthcare organizations.

And just understanding what we’re trying to do here, sometimes it’s hard for outsiders, and I notice that when I talk with my own students it’s very hard to understand, and it takes them about two weeks to kind of get a grasp on what our clinic is all about and how are our clients different. (dietician)

In one clinic, this sense of operating in isolation extended to participants’ sense that the board of directors, who were ostensibly providing oversight of their clinic, understood poorly the nature of their work. Providing care to populations marginalized by poverty, discrimination and other forms of disadvantage, they felt marginalized themselves by the larger structures of the healthcare system.

At times I felt very much like a lone wolf, I mean this is a very conservative community […] even at the board level, how do you let them know that this is the organization they’re running? (nurse practitioner)

The normalized dominance of biomedical approaches to primary health care and efficiency discourses manifest in performance indicators created tensions between the clinics’ commitments to health equity and the mainstream indicators of healthcare quality. Required use of mainstream quality indicators in evaluation and funding processes further pushed the organizations at the margins. Participants described how efficiency-enhancing approaches to health care management, for example rigid appointment scheduling, was at odds with the philosophy of the clinic.

You still have to see X number of clients in a day in order to make the bean counters happy. And you still, you know, you still have all these, these organizational pressures that you have to go into. (nurse practitioner)

Some participants recounted challenges meeting accountability criteria of their professional associations, grounded in a biomedical and egalitarian perspective of healthcare, which is often difficult to reconcile with, for example, harm reduction approaches that are part of EOHC. For example, physicians debated with their professional and regulatory associations about their opioid prescribing practices for “patients with addictions.” Tensions among differing approaches to opioid prescribing practices mirrored discourses about abstinence versus harm reduction approaches, and often remained unresolved, with detrimental impacts for patients, and persistent frustration among providers.

Which of these patients are palliative and which of these patients are addiction? And my response is that’s not how I would classify these patients. I have patients who, on this list, I’ve prescribed opiates to who are palliative, yes, and there are patients on this list that have chronic pain and I’ve prescribed opiates to. Do some of them also have addiction issues, yes, (…) someone comes in with diabetes and say oh I’m not gonna treat your hypertension because I’m gonna focus on your diabetes. Just because someone comes in with a history of addictions doesn’t mean we can just say, we can’t treat your pain. I feel strongly about that. (physician)

Participants also felt excluded in material ways. Being the “other” in the healthcare system compromised their ability to tap into resources that were ostensibly available outside the clinic. For example, many highlighted the rigidity of mainstream healthcare structures and policies that impose limitations on providers’ abilities to refer patients to needed services.

To refer to someone in the pain clinic at the hospital is almost impossible, the amount of documentation, referrals and all that it takes is just insurmountable. And then they don’t want to treat anybody with severe mental health or substance use disorders so you can cross off our entire patient population. (nurse)

About the institutional integration of equity, how do you start to get into these mammoth structures that are hard to change (…), and how the hospital manages people who don’t fit into their nice neat ticky boxes. (physician)

Being positioned structurally at the margins of the mainstream healthcare system translated into the individual level of practice, influencing healthcare providers to push equity to the margins of their day-to-day practice.

### Discourses align with structures and policies to position equity at the margins of health care

Throughout the interviews, participants emphasized the importance of equity. Even though they understood what was needed to provide EOHC and were employed in organizations that were focused on providing care to populations marginalized by poverty, discrimination and other forms of disadvantage, many participants had a compartmentalized perspective of their professional role regarding health equity. They separated their commitment to health equity and their professional role, considering equity as an “extra” element of care, which contributed to pushing it to the margins of their day-to-day practice. The following excerpt illustrates how trauma-informed care, which is a component of EOHC, was not considered central to participants’ professional roles and identities, thus, marginalizing equity.

You know it’s hard to parse out something like trauma-informed care when I’m just delivering care in general. (nurse)

This marginality of equity was also visible in the language participants used to describe their practice, contributing to conceptualizing equity as an “extra” to usual practice. Although participants acknowledged that their everyday practice was inspired by equity principles, they rarely described their approach of care as being equity-oriented. “Equity (…) we do it and we all know that we do it but it’s just kind of naming it.”(counsellor).

Most participants explained that providing EOHC required doing things differently to go beyond individual interactions and to tackle privilege, trauma, and historical contexts in healthcare. For example, to provide EOHC, participants argued that it was essential to acknowledge that trauma is a background context of care.

The point again that really sticks to me is the idea about not treating trauma but using it as a context (…) as a younger nurse you tend to have ideas that you’re going to solve people’s problems (…) as you experience, you realize that most of what people are going through is outside of the scope of what you’re able to, to do in terms of “solving problems”. And that, it’s more important to support people wherever they’re at or what they’re doing. (nurse)

Seeing equity as beyond their scope was not merely the perspective of individual providers, but rather was embedded within organizational policies and how care delivery is structured. Thus, in concert with siloed professional designations and identities, equity was pushed to the margins by organizational practices. Participants underlined that equity is not part of initial health professionals’ training, “we have a lot to learn because we don’t particularly learn it in school” (counsellor). Training related to equity was mostly accessed through self-learning on their own personal time, contributing to pushing equity further to the margins of practice. Participants stated the importance of training to better articulate, name and operationalize EOHC.

Some participants also felt a disconnection between administrative and clinical staff. Participants said that to tailor care to individual patients’ circumstances they had to “drop the usual guidelines” that were based on efficiency indicators and grounded in egalitarian perspectives. Even when the clinic philosophy explicitly stated equity as a priority, participants described how work was still needed at all levels of the organization to be more fully supported fulfilling this commitment.

I think it has to start at the top and it has to be led by example and the people that are at the top need to be making it a priority to walk the talk and not just talk it. And for me I think it really does start at the top and that means our board, that means our executive director, public healthcare coordinator those people need to be leading by example and often what you see is a real disconnect between the people that do the work and the people that are in charge. (administrator)

Structures and practices worked in concert to push equity to the margins of healthcare despite the commitment to equity from both clinics and participants. A perceived lack of organizational support to provide EOHC, conceptualizations of professional roles and identities, and organizational practices all contributed. To harness broader discourses and circumvent barriers to EOHC healthcare providers and organizations developed hybrid approaches to care.

### Staff and organizations navigate competing discourses through hybrid approaches to care

While striving to provide EOHC, participants interacted daily with broader biomedical, culturalist, racializing, efficiency, and egalitarian discourses. Dealing with the contradictions and tensions among these discourses on the one hand, and their commitments to equity on the other, led to the implementation of hybrid approaches to care. Thus, when working to operationalize equity in practice, their approach to care was not only equity-oriented but rather, reflected a complex combination of components of various discourses.

This hybrid approach developed in the context of the pervasiveness of neoliberal and racializing discourses. Racializing discourses continued to influence aspects of some participants’ language and thought patterns. In this excerpt, there is a taken-for-granted assumption that tailoring care requires being sensitive to, or attention to, race-based characteristics, in this case the notion that eye contact is a racial characteristic rather than a strategy for dealing with power dynamics.

[…] we all think that we're sensitive to racial differences. However, we might not be as sensitive as we think just in terms of, like, looking people directly in the eye and how one race would look at that differently. (administrator)

Even when the staff members realized the importance of continuously examining unconscious biases, racializing and culturalist discourses persisted, and staff often struggled to find the language to frame the issues they faced at the point of care, often reverting to culturalist, race-based thinking:

All of us need to keep revisiting this [racializing behaviour] because a lot of it is unconscious, however, you have to be aware of every different nationality or culture that comes in here. (administrator)

I think that it would have been really helpful to have someone from the Indigenous community come in and teach how their culture would do it anyway (medical office assistant)

Thus, even in organizations with explicit commitments to health equity, dominant racializing and culturalist discourses are pervasive and can affect the attitudes and practices of staff members, and in some cases, create harm for patients. In the following excerpt, a participant working at an administrative level reflected on an interaction between a clinician and an Indigenous patient. Even as the clinician struggled to support a better response, she continued to reflect assumptions that link Indigenous peoples to “drug seeking” behaviours – assumptions that result in negative judgments and ongoing stigma toward Indigenous people [13].

We had someone who felt they were being judged because they were Aboriginal because the [provider’s] discussion [with the patient] centred around, “do you know that nurse practitioners can’t prescribe narcotics?” God why did you say that? So we rehearsed, I said so here’s how I would have handled that. I would have started with “as long as you understand that you’re welcome in the practice but we can’t prescribe controlled substances”. I said that’s the word I use now (…) You didn’t think you were making a judgment but they heard a judgment. And maybe you can’t own that and that’s okay but I think we need to also look at what helped them hear that (…) and I think it was the word narcotic. (clinical lead)

Although the participant acknowledged that making an association between drug use and being an Indigenous person is problematic, this same association is evident in her suggested revised response. She focuses on whether the words “substance” or “narcotic” should be used rather than on the harmful association. Similarly, a counsellor from another clinic implicitly associated drug and alcohol use with being Indigenous, also obscuring the potential influence of racialized power dynamics on being “reluctant to talk.”

I think maybe again I talk a lot about this is just that awareness now of, of seeing that maybe the work I do with First Nation’s people and the work I do with say people who don’t have a drug or an alcohol problem and they’re in here for an entirely different reason is that my approach with them is very consistent. I will be more aware maybe that the First Nations might be reluctant to talk…as opposed to say somebody who doesn’t have an alcohol or drug addiction in their past. (counsellor)

Tailoring care to contexts was also sometimes conflated with a culturalist approach of care. Speaking about the online Indigenous Cultural Safety^1^ training that was taken as part of the EQUIP intervention, one participant expressed dissatisfaction related to the fact that the training did not focus on cultural characteristics of Indigenous peoples. In the following excerpts, there is an assumption that culture is associated with specific behaviours and practices common among a group of persons.

I felt like I learned nothing about the culture of First Nation’s people beyond the trauma that they’ve gone through. (…) there should have been more focus on their culture, the positive aspects of their culture and who they are, how their family systems are organized and their spiritual practice and their food (…) Yeah, I would have liked to have learned more about that as well as because it seems like there was a real lack of learning about their culture. (medical office assistant)

A hybrid approach was evident in how providers’ practices simultaneously resisted and colluded with discourses that contradict their goals of equity. Efficiency goals, role definitions and professional identities combined to support and reinforce the dominant biomedical approaches to care which participants saw as contradicting their intentions toward equity, creating challenges in their day-to-day practice.

How can I help someone work through their chronic pain given the current, the tool set that I have? And how do I change the tool set within […] that whole systematic, the institutional, the way mental health is organized, the way medicine is organized in the current structures that makes it more difficult. (physician)

Nevertheless, participants worked to circumvent some difficulties related to biomedical approaches to orient their care toward equity. In one clinic, for example, one participant described making the relationship with the patient the top priority instead of the medical act itself.

In the end I think it’s all about the relationship. And that it’s something that I think sometimes gets overlooked is the importance of taking the time to build the relationship (…) And so when you have a physician churning people through in five minutes that’s not going to be relationship building (…) every patient who loves their doctor it’s because it’s the doctors who spend time with them and listen to them and that’s what it’s about so maybe the tension isn’t so much about prescribing but it’s about ways of being… (nurse)

In addition, having an equity-informed understanding of the population served by the clinics helped participants to navigate pervasive efficiency discourses and impelled personal and structural changes toward a more context-responsive approach congruent with EOHC. For example, population statistics collected at the clinic level were used to legitimate or find funding for activities to translate the clinics’ commitment to health equity into programs and practices. Statistics were also used to measure the effectiveness of interventions and programs at the clinics and reorient them, if needed, toward the clinics’ philosophy.

I started pulling out data to figure out how many kids are coming into care. So that’s when I kind of looked at the data and we saw, wow, we have a lot of kids here we actually don’t see hardly any of them. (…) So we made a safe space [for children in the clinic] and it wasn’t just me who was like let’s put this space here, it was everybody. (nurse practitioner)

And then how and when to use that like that’s really good data that we can use for maybe more funding to get more alcohol and drug counsellors, mental health counsellors, physio because, you know, trauma comes out in physical pain that kind of stuff. So I think those numbers are really valuable to take to [the health authority] and say hey, we need more money, we need more staff. (nurse)

Considering the pervasiveness of multiple discourses influencing the practices of the staff at the clinics and the fact that EOHC is in constant evolution, participants raised the importance of continuously reflecting on their practices to challenge discourses competing with the clinics’ commitment to health equity and use them in the service of health equity.

We always have ongoing discussions on how to make us more relevant, make this place more culturally safe and relevant for our clients. (nurse)

The multiple discourses encountered in providers’ day-to-day practice gave them the opportunity to reflect on their practice and to develop an approach that they intend to be oriented toward equity.

## Discussion

Results of this analysis highlight the need for providers and organizations to continuously examine pervasive efficiency, racializing and culturalist discourses and how they are playing out in the health care context. In Canada, an innovative program grounded in anti-racist pedagogy, critical race theory, and transformative learning principles, known as the San’yas Indigenous Cultural Safety training, has been helpful in enhancing responsiveness to racism and stereotyping generally [32, 36]. This type of training could be instrumental in building health care staff and administrators’ awareness of dominant culturalist and racializing discourses and enhance their capacity to challenge theses discourses at the organizational level. However, for interventions to be disruptive and challenge the status quo, new organizational structures and processes are also necessary to manage the shifts in power dynamics and taken-for-granted practices [32, 37].

As shown in the results of this study, organizations such as primary health care clinics must have the capacity to respond to competing demands from the mainstream healthcare system and funders [30], their communities and health care providers. These demands, which are variously guided and influenced by both EOHC principles, and efficiency and accountability requirements, are often difficult to reconcile. At the same time, studies show that discourses of efficiency and accountability can be mobilized as goals in the service of EOHC. For example, in prior research, Browne et al. [13] showed that efficiency can be achieved using an EOHC approach comprising 10 strategies that intersect to optimize effectiveness of healthcare services for Indigenous peoples. New evidence suggests that equity-oriented care is associated with positive health outcomes for people experiencing health and social inequities, and that interventions delivered at the point-of-care, along with supportive organizational leadership, can improve providers’ knowledge and confidence related to providing such care [32, 33]. In this regard, workplace learning initiatives aimed at both professional development and organizational change represent a promising avenue for the promotion of EOHC. EOHC could be supported and sustained by staff forming communities of practice dedicated to health equity and anti-discrimination. Communities of practice represent a collaborative interprofessional learning strategy [38] and have been very successful in increasing a sense of ownership and autonomy among participants [39]. They can benefit the individual, a team of co-workers, and the organization [39, 40] and are considered dynamic adaptive systems that evolve over time within the organizational context and social environment [41]. This may be an effective, sustainable, and low-cost strategy for organizations – enabling them to continually revisit, challenge, and respond to competing discourses in their individual, institutional, and systemic forms.

Results of this study also highlight that the responsibility for change toward health equity cannot be delegated only to point-of-care workers. Indeed, despite mandates and commitments to health equity, the clinics in this study were structured to align with mainstream health care system goals and priorities – dictated by funding priorities, performance indicators, and policies emanating from the healthcare system, professional organizations, and rules governing the organization within which the clinics were embedded [30]. This points to a need for mainstream agencies to make fundamental changes to their service-delivery systems and the dominant values underpinning their practices, such as equality and universalism. For example, the emphasis could shift from the so-called universal approach of mainstream services toward services that are more tailored to meet the needs of individuals and communities experiencing inequities and having the poorest health outcomes. In that sense, patients’ perspectives rarely inform decisions about policies and practices at health centres. Considering underserved patients’ voices seems to be an untapped potential. Thus, instead of considering these patients’ experiences as exceptions that need to be accommodated on an ad hoc basis, approaches for addressing the most pressing healthcare needs could be integrated into the structures, policies, programs, and practices of mainstream healthcare systems; these approaches could be considered “the norm” for good quality and equitable care for all.

## Conclusion

This study highlights the importance of making visible the ways in which competing discourses influence, reinforce, and challenge current practices, and to take action so these dynamics can be enlisted to promote health equity at the clinical, organizational, and systemic levels. It is time to develop strategies to help shift health equity from being an “add-on” to usual care, to being an integral and fundamental aspect of care. By naming equity and providing tools to set equity as an expectation of good-quality care, this “extra” practice can become part of the role and responsibility of everybody working in primary health care and other healthcare settings, from receptionists to board members and government leaders. Considering equity as everyone’s responsibility in the day-to-day life of organizations and systems has the potential to prepare people to tackle structural constraints to health equity and position EOHC at the centre of healthcare practices.

## Data Availability

The datasets used/or analyzed during the current study are available from the corresponding author on a reasonable request.

## Abbreviations

CDA: Critical discourse analysis
ED: Emergency departments
EOHC: Equity-oriented health care
EQUIP: Equipping Primary Health Care for Equity
MOA: Medical office assistant
NHS: National Health Services
PHC: Primary health care

## Footnotes

1 The San'yas Indigenous Cultural Safety training program was developed in British Columbia, Canada by the Provincial Health Services Authority Aboriginal Health Program. It is a facilitated online training program designed to increase understanding of Indigenous people, and knowledge, self-awareness, and skills of those working directly or indirectly with Indigenous people (http://www.sanyas.ca/).
